# Supplementary material for: Tweet for Behavior Change: Using Social Media for the Dissemination of Public Health Messages
Source: JMIR Public Health Surveill. 2017 Mar 23;3(1):e14. doi: 10.2196/publichealth.6313 (PMC5383801; doi:10.2196/publichealth.6313)
Supplement: Multimedia Appendix 1 [file publichealth_v3i1e14_app1.pdf]

Appendix I: Key word search terms

(1) General
Care in the Sun
Sun Safety
Sun Safe Behaviour
Tanning
Sunbeds / Tanning Beds / Indoor Tanning / Tanning Salon
UV Exposure
Sunglasses
Sun Exposure
Sunscreen / Sun Cream / Sun Block
Sunless Tanning (spray tan, self tan, tanning lotion, fake tan)

(2) Health condition and sun-related effects on human skin
Melanoma
Carcinoma
Basal Cell
Squamous Cell
Skin Neoplasm
Sunburn Related (-sunburn, burn, pain, itch, danger, hurt, ouch, ow)
Skin Aging
Wrinkles/Ageing
Suntan
Skin Cancer / Sun Cancer
Tanning
Skin Deep
Moles
Vitamin D

(3) Type of interventions, as well as main behavioural and social cognitive outcomes
Health Promotion
Health Behaviour
Public Health
Attitude
Knowledge
Prevention of Skin Cancer
Checking your Skin
Checking Moles
Healthy Tan
UV Protection

(4) Recreational settings
Recreation
Tourism
Holidays
Bathing Beaches
Swimming Pools
Outdoor Activity
Sport
Working Outdoors
