# Supplementary material for: Tweet for Behavior Change: Using Social Media for the Dissemination of Public Health Messages
Source: JMIR Public Health Surveill. 2017 Mar 23;3(1):e14. doi: 10.2196/publichealth.6313 (PMC5383801; doi:10.2196/publichealth.6313)
Supplement: Multimedia Appendix 2 [file publichealth_v3i1e14_app2.pdf]

Appendix II: Intervention Development

Firstly, a review of the literature on intervention studies related to skin cancer prevention was conducted to ascertain effective Behaviour Change Techniques (BCTs), intervention components and their theoretical underpinnings, and public health reviews and guidance was consulted (e.g. from NICE/CRUK).

Secondly, a representative Northern Ireland household survey (n=752) was externally commissioned in April 2015, via face-to-face interviews in people's homes by trained interviewers to identify opportunities for the intervention, particularly social media use, familiarity with existing campaigns and knowledge and attitudes relating to UV exposure and skin cancer prevention, and also to provide broader views including from those who may not necessarily use social media. The survey used a quota-based sample, stratified by postcode to provide a representative distribution across NI.

Focus groups were also undertaken with students (n=6) and stakeholders (n=15) to explore their views on the design, plausibility and acceptability of the social media campaign for the promotion of skin health and care in the sun. Focus group discussions also explored views on the potential content and implementation of the campaign, access and privacy issues relating to its implementation and a logic model for effective delivery. Participants were recruited through our Patient and Public Involvement (PPI) networks, public health professionals and social marketing advisors who work with our partners, the Public Health Agency, the regional cancer charity and academics with expertise in health psychology, risk communication and public health. Using this information, a "co-design" workshop, modelled on the 'Future Workshop' methodology [1] was undertaken to give participants (n=15) the opportunity to critique existing materials and to participate in designing the elements of a prototype social media intervention. Discussions from both the focus groups and the co-design workshop contributed to the development of a Logic Model (Appendix IV), from which the intervention was developed.

Strategies were developed to aid dissemination and public engagement. These strategies included the use of opportunistic messages (Figure 1), which are those relating to popular public events such as the local Marathon, the regional Agricultural Show or which mention the local weather on a given day. Another strategy was the recruitment of 'seeds' or influencers with a notable social media following, including local TV weather presenters, local sports clubs and musicians who were contacted and invited to retweet campaign content to their followers, with the aim of ultimately creating viral health messages.

Message frames and sentiment were also explored via focus groups and in the co-design workshop. After reviewing existing campaign content utilised by bodies such as Cancer Research UK and in the 'Slip, Slop, Slap' campaign, five key message types were identified for the campaign. These were categorised as: informative; personal stories; shock/disgust; humorous and opportunistic. Informative messages, denoted by the hashtag #info, were those which educated the public about skin cancer risks and how to check their skin. Personal story messages were those which documented a person's experience of skin cancer (#story). The hashtag '#eek' was identified to denote shock or disgust messages, such as images which would be considered uncomfortable to view or shocking statistics relating to skin cancer. Humorous messages included "internet memes" and funny or sarcastic images aimed at promoting skin cancer awareness (#geg [colloquial term for humour in NI]). Hashtags were chosen to be meaningful to the local population. For example, 'geg' would be recognised by the Northern Ireland population as vernacular to denote humour. Examples of the message types are displayed in Figure 1.

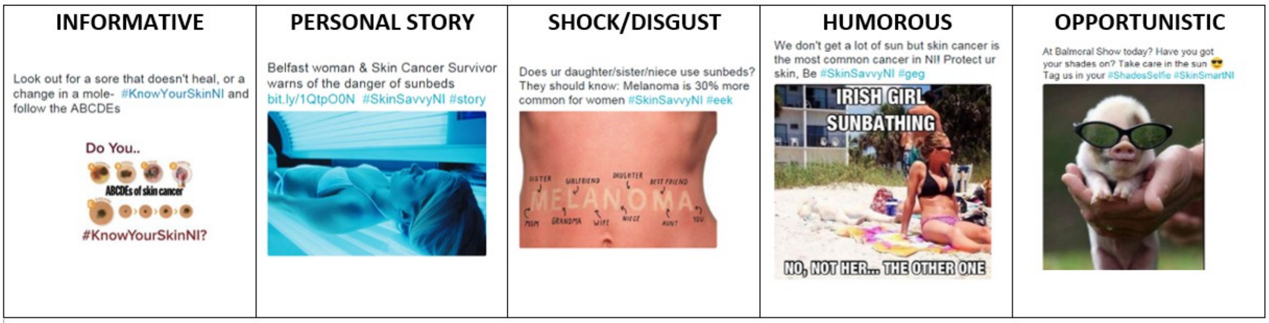

Figure 1: Examples of message types used during the intervention

Reference:

1. Müllert N, Jungk R. Future Workshops: How to create desirable futures. London, United Kingdom: Institute for Social Inventions, London (United Kingdom). 1987.
