# Supplementary material for: Tweet for Behavior Change: Using Social Media for the Dissemination of Public Health Messages
Source: JMIR Public Health Surveill. 2017 Mar 23;3(1):e14. doi: 10.2196/publichealth.6313 (PMC5383801; doi:10.2196/publichealth.6313)
Supplement: Multimedia Appendix 3 [file publichealth_v3i1e14_app3.pdf]

Appendix III: Checklist for Reporting Results of Internet E-Surveys (CHERRIES)

	**Checklist for Reporting Results of Internet E-Surveys (CHERRIES)**
Item Category	***Checklist Item and Explanation***
**Design**	The e-surveys of the present study were aimed to collect data from adults across Northern Ireland. The surveys were administered both pre, and post-intervention.
**IRB (Institutional Review Board) approval and informed consent process**	Approval was sought and granted by the Research Ethics Committee of the School of Medicine, Dentistry and Biomedical Sciences, Queen's University Belfast, Northern Ireland (Ref 14/55).<br><br>Regarding informed consent, participants were provided with details regarding the length of time of the survey, data storage, the purpose of the study and the organisations responsible for it.<br><br>Data was stored appropriately following University guidance.
**Development and pre-testing**	The surveys were designed, as an open survey using Qualtrics. The measures were modelled on the Cancer Research UK SunSmart survey [1]. The target population was adults (aged >18 years) living in Northern Ireland. The surveys were pre-tested for usability and functionality and piloted amongst the research team.
**Recruitment process and description of the sample having access to the questionnaire**	An advertisement was placed on social media inviting adults aged >18 years in Northern Ireland to participate in a survey for the chance to win an iPad Mini. Those who clicked on the advertisement were redirected to a Qualtrics website survey. Paid-for 'promoted' tweets were used to reach a wider audience (Appendix V).<br><br>The following text was used at the beginning of the survey to instruct participants:<br>"There are 3 sections in the questionnaire. Please answer all questions in each section as instructed. However, if you do not wish to answer a question(s) in any section just leave the response blank."<br><br>Participants had the option to go back to review/change answers.<br>Blank responses were coded as missing.<br><br>IP addresses were logged and exported with the survey data.<br>Participation rates were measured based on those who started the survey, and those who were included in analyses, as a number of completers were excluded based on age and/or location. The participation rate for the pre-intervention survey was 67.5% and 66.2% post-intervention
**Analysis**	Data was analysed for completed questionnaires and those which completed the final section of the questionnaire.<br>Whilst time-stamps were recorded, this was not used as a cut-off point.

References

1. Miles A, Waller J, Hiom S, Swanston D. SunSmart? Skin cancer knowledge and preventive behaviour in a British population representative sample. Health Education Research. 2005 Oct 1;20(5):579-85.
