# Supplementary material for: Tweet for Behavior Change: Using Social Media for the Dissemination of Public Health Messages
Source: JMIR Public Health Surveill. 2017 Mar 23;3(1):e14. doi: 10.2196/publichealth.6313 (PMC5383801; doi:10.2196/publichealth.6313)

Appendix V: Survey advertisement on Twitter

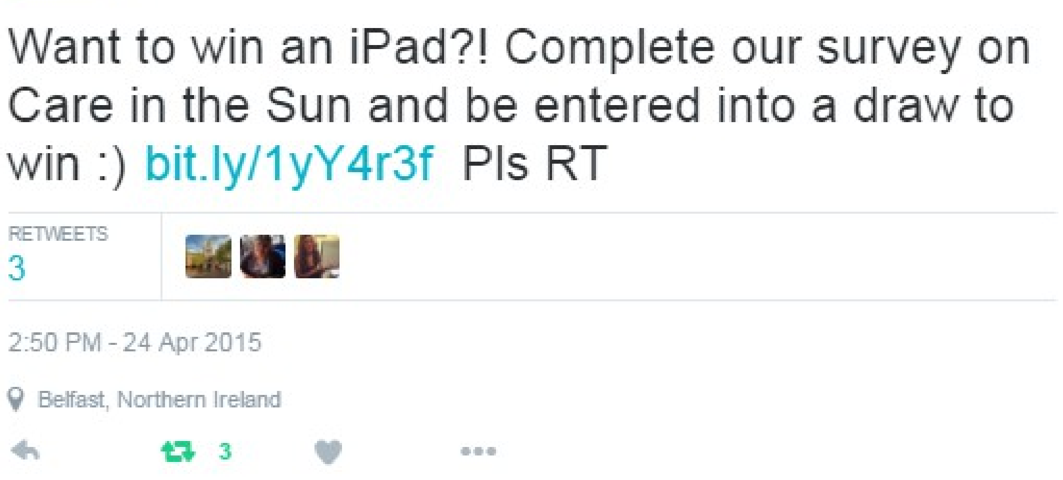

Supplement: Multimedia Appendix 4 [file publichealth_v3i1e14_app4.pdf]
